# Uncovering Shared Diagnostic Genes and Mechanisms in Chronic Kidney Disease and Atherosclerosis: A Bioinformatics Approach

**DOI:** 10.2174/0109298673432178260429092542

**Published:** 2026-05-20

**Authors:** Hua Li, Jinfeng Guo, Hao Luo, Rui Huang, Hongwei Su

**Affiliations:** 1 College of Integrated Traditional and Western Medicine, Southwest Medical University, Luzhou, 646000, China;; 2 Department of Urology, The Affiliated Traditional Medicine Hospital, Southwest Medical University, Luzhou, 646000, China

**Keywords:** Chronic kidney disease, atherosclerosis, bioinformatics, machine learning, Gene Expression Omnibus (GEO), NF-kappa B

## Abstract

**Objective:**

The purpose of this study was to screen for common diagnostic genes and identify possible molecular mechanisms underlying CKD combined with AS using bioinformatic methods.

**Methods:**

We retrieved the gene expression profiles of CKD and AS from the Gene Expression Omnibus (GEO). Using differential expression combined with WGCNA, we identified hub genes shared by CKD and AS. GO and KEGG enrichment analyses were conducted. To screen the diagnostic biomarker, we constructed PPI networks and applied three ML methods: least absolute shrinkage and selection operator (ASSO), random forest (RF), and Support Vector Machine-Recursive Feature Elimination (SVM-RFE). The diagnostic performance for the candidate gene was assessed by a receiver operating characteristic curve (ROC) on the training set and the external validation dataset. CIBERSORT is used to evaluate immune cell infiltration the DGIdb database predicts possible drugs for treatment.

**Results:**

We found a total of 119 common genes highly correlated between CKD and AS. The enrichment results showed that these genes are mainly related to the cytoskeleton in the muscle cell, vascular smooth muscle contraction, cGMP-PKG signaling pathway and NF-kappa B signaling pathway. By the integrative PPI and ML analysis, TAGLN was selected as the hub common gene. TAGLN had good diagnostic efficiency (AUC > 0.70) across the training and validation cohorts. Immune infiltration analysis showed significant dysregulation of several immune cells for both diseases. Moreover, two candidate drugs acting on TAGLN were found.

**Discussion:**

In this research, we have revealed that TAGLN was an effective diagnostic indicator in CKD-AS comorbidity. The multi-algorithms confirmed TAGLN’s outstanding diagnostic ability among six independent samples. Mechanistically, cytoskeletal dysregulation seems to be the key connection of the two diseases, and there is a strong correlation between TAGLN expression and immune cell infiltration. Recombinant transforming growth factor-β1 and azacitidine could possibly function by modulating TAGLN. Our study not only reveals the molecular basis of CKD-AS comorbidity but also provides some useful targets for developing novel diagnostic or therapeutic strategies.

**Conclusion:**

We believe our results provide clues to the possibility of using TAGLN as an ideal common diagnostic marker for CKD and AS, and point out some common molecular mechanisms and possible treatment targets between the two diseases that will provide new clues to future research on their pathogenesis, diagnosis, and therapy.

## 1 INTRODUCTION

CKD refers to persistent abnormalities in kidney structure or function lasting at least three months, which adversely affect overall health [1]. CKD may progress into kidney failure or ESRD and increases the risks of CVDs with increasing severity over time; a recent report from the Global Burden of Disease group ranked CKD among the top 10 causes for adverse health outcomes globally (in 2020) [2]. Moreover, a meta-analysis of observational studies showed that around 13.4% of the world’s population suffers from CKD [3]. Complications related to CKD can lead to faster progression of the disease and increased risk of cardiovascular-related morbidities [4]. AS is a chronic, polyfocal, immuno-inflammatory process of the vessels with involvement of medium-sized and large arteries, which is predominantly caused by lipids. Atherosclerosis is a major cause of diseases like coronary artery disease, carotid artery disease, and peripheral arterial disease [5]. The primary lesions associated with AS are characterized by lipid accumulation within arterial walls, along with the proliferation of smooth muscle cells and fibrous tissue, ultimately leading to the formation of atherosclerotic plaques [6]. AS serves as the pathological basis for cardiovascular disease, where the rupture of unstable atherosclerotic plaques, coupled with platelet aggregation and thrombosis, can result in vascular narrowing or blockage, precipitating acute cardiovascular events [7, 8].

AS is often seen in patients with chronic kidney disease CKD. AS and its manifestation in the coronal vessels are regarded as important links between CKD and CV morbidities and mortalities. It has been proven that renal insufficiency relates to the severity of coronary atherosclerosis, the risk of coronary events, and mortality after a myocardial infarction [9-11]. Thus, it is important to identify relevant factors and common pathophysiological pathways that lead to concomitant development of CKD and AS.

As modern biotechnology develops, bioinformatics analysis provides an effective way for investigating the underlying mechanisms and candidate biomarkers of multiple diseases. In our study, we used integrated bioinformatics methods for analyzing common genes and related mechanisms between CKD and AS, then analyzed common genes by ROC curve and immunoinfiltration. Finally, we validated candidate drug targets in these two diseases. This study can offer some novel clues about the common pathogenesis of CKD and AS, providing essential data support and chances to make breakthroughs on their joint prevention and treatment. The outline map of the research process is shown as follows, see Fig. (**1**).

## 2 MATERIALS AND METHODS DATA SOURCES

GEO is a public functional genomic data repository that holds many of the publicly accessible gene expression data sources from where scientists can aggregate them and perform analysis on them [12, 13]. We use the gene expression data sets collected here. We took two data sets (GSE100927 [14] and GSE66494 [15]) as the training set, in which there are 69 arterial tissues containing atherosclerotic lesion and 35 tissues free of atherosclerotic lesions in GSE100927, and GSE66494 has 53 chronic kidney disease and 8 normal kidney tissues’ biopsy samples; Four independent datasets were also used for the validation cohorts: GSE43292 [16] (32 atherosclerotic plaques *vs.* GSM348781-GSM348813 (n=32 remote intact tissue samples); GSE57691 [17] (n=9 aortic occlusive disease, n=10 control); GSE142153 [18] (n=7 peripheral blood of end-stage renal disease (ESRD) subjects and n=10 peripheral blood of normal volunteers); and GSE37171 [19] (whole blood of 75 chronic renal failure patients *vs.* 40 healthy controls).

### 2.1 Differentially Expressed Genes Analysis

The DEG analysis was performed by the “limma” package in R software. In order to get an ideal size of the DGEs dataset, we set the screen threshold of CKD(GSE66494) as “adjust *p* < 0.05, |logFC| > 0.75”. [19], and the cutoff of screen for AS (GSE100927) is adjusted *p* < 0.05, |logFC| > 0.5 [20].Then we used the “ggplot2” package to generate volcano plots for visualizing upregulated and downregulated DEGs, and the “pheatmap” package to generate heatmaps for visualizing the most differentially expressed (top 30) genes. In addition, a Venn map was built in the bioinformatics website (https://www.bioinformatics.com.cn) [21] for visualization of the shared DEGs from CKD and AS, and we called this intersection set of genes “DEGs”.

### 2.2 Weighted Gene Co-expression Network Analysis

The WGCNA [22] analysis was carried out based on the R package in order to find gene modules related to AS and CKD, which built up a scale-free co-expression network *via* raising the pairwise Pearson’s correlation matrix of genes to some soft thresholding power (β), was chosen as the smallest value for which the R2 value of the scale-free topology fit was greater than or equal to 0.90 [23]. The resulting adjacency matrix was transformed into a TOM that provides a more stable representation of biological similarity. We used dynamic tree cut to identify gene modules from the TOM-based dissimilarity matrix, where we set the min module size as 50 [24], deepSplit of 2 [25], and a merge cut height of 0.25 [26], merging modules whose eigengene correlations are >0.75. Module-trait relationships were evaluated by correlating the MEs to clinical traits, using a threshold for clinical relevance where modules were defined to have correlations with |r| >0.5, *p*<0.05 [27]. We collected all the important genes in AS analysis and CKD analysis as “CKD_module” and “AS_module” data sets, respectively. We merged these two modules together and removed duplicates from the Module genes data set. Then, the common genes in AS and CKD were calculated using “DEGs” and “Module genes”, and a Venn map was plotted to show them.

### 2.3 Functional Enrichment Analysis

The functional enrichment analysis of the common genes for GO terms and KEGG pathways was conducted by using the Database for Annotation, Visualization and Integrated Discovery (DAVID) bioinformatics resources(https://davidbioinformatics.nih.gov) [28]. Gene Ontology (GO) offers a controlled vocabulary to describe gene product function in three main aspects: molecular function and component, whereas KEGG enables the structured annotation of gene activity in known signalling and metabolic pathways [29, 30]. The top 8 enriched GO terms and the top 12 significant KEGG pathways were graphically summarized by the bioinformatics platform.

### 2.4 Construction of the PPI Network and Identification of Core Genes

The common genes were queried in the STRING database (https://string-db.org) to build a PPI network, where the species is set as Homo sapiens, and the minimum interaction confidence is > 0.4 [31]. Then it was imported to Cytoscape to visualize and analyze. The hub genes in this network were found based on the CytoHubba plug-in through five different algorithms: Degree, MCC (Maximal Clique Centrality), Density of Maximum Neighborhood Component (DMNC), Edge Percolated Component (EPC), and Maximum Neighborhood Component (MNC). The top 10 genes from each algorithm, and overlapping genes in each pair of the five approaches, were considered as the intersection genes; then, the intersection gene set of all five methods was taken as the core hub genes. The overlaps were visualized by our own written Venn program.

### 2.5 Candidate Biomarker Identification Through Machine Learning

In order to optimize this method of selecting genes and find common candidate diagnostic markers in AS and CKD, we selected these core genes that were pre-selected for further research. Finally, a machine learning algorithm combining multiple models (LASSO), RF, and SVM-RFE [32-34]. The LASSO model was built, and predictors were screened by using the “glmnet” package in R software. The advantage of LASSO is that it can be used for selecting variables to reduce model complexity with a series of parameters, thus effectively preventing over-fitting. We performed a 10- fold cross-validation to compute the lambda (λ) values in R; then we took the λ value with the lowest error as our criterion for screening the predictors [35, 36]. Meanwhile, we also used the ‘randomForest’ library of R to build an RF classifier and set the number of trees as ntrees=500. We assessed feature importance by using the mean decrease of the Gini index (MDG). We computed the MDG value for all features, then sorted them in a descending manner, where the genes that have a larger MDG are deemed to be of greater biological relevance [37, 38]. At the same time, we applied the SVM-RFE algorithm for iterative selection of features that are more informative [6]. The analysis was performed using the package “e1071” from the R software. For a linear kernel, the procedure iteratively removed the feature with the lowest squared weight coefficient of that step, and a 5-fold cross-validation was included to ensure model stability in every iteration, ultimately producing a ranked list of all features [39-41]. Finally, the intersecting genes identified consistently across all three machine learning methods were established as candidate biomarkers for both AS and CKD.

### 2.6 Diagnostic Evaluation of Candidate Biomarkers

To assess the diagnostic power of this candidate biomarker, we conducted ROC curve analyses for both training and validation data sets by utilizing the “pROC” R package [42]. The closer to the top-left angle the curve is, the higher the true-positive rate and the lower the false-positive rate it has, reflecting better diagnostic performance. We use the AUC as a numerical measure and consider it to be significant if it is larger than 0.7 in two cohorts, suggesting that there is a good diagnostic power for candidate genes to screen AS and CKD [43].

### 2.7 Immune Infiltration Analysis

The immune infiltration of the screened diagnostic biomarker was analyzed by the CIBERSORT algorithm(http://cibersort.stanford.edu/) [44] and then plotted, and further correlation analysis was performed by means of the ”pheatmap”, ”ggplot2”, ”corrplot”, and ”vioplot” packages from R [45]. For that purpose, Spearman correlations were computed for each of the immune cell types with respect to the biomarker.

### 2.8 Drug Prediction

We used the Drug-Gene Interaction Database (DGIdb, www.dgidb.org) to predict putative drug interactions of these discovered diagnostic biomarkers [46]. DGIdb is a compendium integrating drug-gene interaction data as well as druggable gene information from various sources, including the literature, existing data sources, or other websites [47].

## 3 RESULTS

### 3.1 Differentially Expressed Genes Screening

DEG screening was obtained by differential gene expression analysis based on cutoffs: |logFC|>0.5 and *P*-value < 0.05 in GSE100927 and |logFC|>0.75 and *P*- value < 0.05 in GSE66494 datasets, respectively. A total of 2192 DEGs were screened out from GSE100927(1257 up-and 935 down- regulated; Fig. **2A**), while 1743 DEGs were obtained from GSE66494 (481 up- and 1262 down- regulated, Fig. **2B**). A heatmap was plotted for the top 30 up- and down-regulated genes in each of these datasets (Fig. **2C**, **D**). Common DEGs between the two datasets after passing the screening criteria, there were 57 common up-regulated and 73 common down-regulated genes, and are depicted as a Venn diagram (Fig. **3A**, **B**). The overlapping 130 DEGs were defined as “DEGs” and used for further analysis.

### 3.2 Dataset Construction of Module Genes

We used WGCNA to find significant modules (*p* < 0.05) of both GSE100927 and GSE66494, named as “AS_module" and "CKD_module", respectively. We chose the best-fitting soft threshold power (β = 5 for AS; β = 11 for CKD), which resulted in a scale-free topology (Fig. **4A**, **B**). The sample clustering was performed by the blockwiseModules function, which produced a cluster dendrogram (Fig. **4C**, **D**) as well as a heatmap for visualizing the association between modules and traits (module-trait relationship heatmap, Fig. **4E**, **F**). The AS_module consisted of three statistically significant modules (turquoise, brown, and blue; Fig. **4E**), and the CKD_module had four (midnight blue, yellow, cyan, and brown; Fig. **4F**). These results indicated the hub gene expression profiles as well as candidate genes associated with AS and CKD. Finally, after integrating all modules into one module and deleting duplicate modules, a “Module genes” set including 3765 genes; and finally, the intersection of these sets (“Module genes”) with previously defined “DEGs”, which resulted in 119 common genes (Fig. **3C**).

### 3.3 Functional Enrichment Analysis Results

GO functional annotation and KEGG pathways enrichment analyses were performed for the common genes to reveal their functions. Regarding BP, these genes were significantly involved in signal transduction, negative regulation of angiogenesis, and apoptosis (Fig. **5A**). In terms of CCs, they were predominantly localised to the cytosol, actin cytoskeleton, and extracellular region (Fig. **5A**). The molecular functions (MF) were all dominated with the same protein binding, protein homodimerization activity and actin filament binding (Fig. **5A**), Further, KEGG pathway enrichment results showed that the common genes were significantly enriched on cytoskeleton pathways of myocytes, vascular smooth muscle contraction, cGMP - PKG signalling pathway, and NF-kappa B signalling pathway (Fig. **5B**).

### 3.4 Construction of the PPI Network and Identification of Core Genes

To clarify the relationship between these common DEGs, we established a PPI network based on the STRING database (Fig. **6A**), which was then input into Cytoscape for further analysis by the CytoHubba plug-in to find out its hub gene. Select the top 10 genes from each algorithm (Degree, DMNC, MCC, EPC, and MNC) (Figs. **6B**-**F**) and visualize their intersection through the Vien diagram (Fig. **6G**) to determine the final core gene set: FLNA, MYH11, CNN1, TAGLN, TPM2, and ACTG2.

### 3.5 Machine Learning Screening Results

In order to further optimize the candidate hub gene, we used these three machine learning methods for analyzing the six core genes of the AS and CKD dataset, respectively. LASSO regression selected four genes in AS ( FLNA, TAGLN, TPM2, ACTG2; Fig. **7A**) and three in CKD (FLNA, TAGLN, ACTG2; Fig. **7B**). Next, the RF analysis identified six genes in AS (TPM2, TAGLN, FLNA, CNN1, ACTG2, MYH11; Fig. **7C**), while there were three common ones in CKD (TAGLN, TPM2, ACTG2; Fig. **7D**). Additionally, three genes were confirmed by SVM-RFE in AS(TPM2, FLNA, TAGLN; Fig. **7E**) and three in CKD (ACTG2, CNN1, TAGLN; Fig. **7F**). Finally, pooling all results together, TAGLN was the only gene common to all analysis confirming its role as the candidate biomarker (Fig. **7G**).

### 3.6 Diagnosis Values Evaluation

The sensitivity, specificity, and area under the curve (AUC) were calculated for each candidate gene to determine its diagnostic power *via* receiver operating characteristic (ROC). For AS, TAGLN had an AUC of 0.854 in the training set (GSE100927), and yielded consistent AUCs of 0.821, 0.767 on two independent validation sets (GSE43292 and GSE57691) (Fig. **8A**-**C**). In CKD, that showed an AUC of 0.950 in the training set (GSE66494), as well as an AUC of 0.779 and 0.753 on external validation sets (GSE142153, GSE37171) (Fig. **8D**-**F**). The above results strongly verify that TAGLN has a good diagnostic ability in various datasets with high generalizability, which can be used to provide potential diagnostic biomarkers for CKD or AS.

### 3.7 Immune Cell Infiltration Analysis

We applied the CIBERSORT algorithm to characterize the immune cell infiltration landscape in AS and CKD. The compositional distribution of 22 immune cells is illustrated with bar plots and heatmaps (Fig. **9A**, **B**). There was a noticeable difference in the proportion of immune cells across disease *vs.* controls. We identified increased frequencies of memory B cells, regulatory T cells (Treg), gamma delta T cells, M0 macrophage and activated mast cell in the AS samples, and lower fractions of naive B cell, plasma cells, resting memory CD4+ T cells, activated NK, monocytes and M1 macrophages, activated DCs and resting MCs (Fig. **9C**). In the CKD samples, increased levels of CD8+ T cells, monocytes and M0 macrophages correlated with decreased levels of resting memory CD4+ T cells, follicular helper T cells, Tregs, and activated NK cells (Fig. **9D**). Particularly interesting was that M0 macrophages and resting memory CD4+ T cells had consistent directions of change between the two diseases; M0 macrophages are up-regulated, whereas resting memory CD4+ T cells were reduced.

Furthermore, we investigated the correlation between different types of immune cells and found that in the AS sample, M0 macrophage correlated negatively with monocyte (r = -0.72), resting memory CD4+ T cells (r =-0.55), activated NK cells (r =-0.64), and plasma (r =- 0.51).

Positive correlations were found between naive B cells with plasma (r=0.55), and resting mast cell/plasma cells (r = 0.58) while neutrophil showed positive correlation with monocytes (r = 0.55), resting memory CD4+ Tcells (r = 0.57), and resting NK cells (r = 0.62) (Fig. **9E**). In CKD specimens, neutrophils correlated strongly positively with resting NK cells (r = 0.86), activated memory CD4+ T cells (r = 0.74) and monocytes (r = 0.75). Monocytes, resting DCs, and resting NK cells are also positively correlated to activated memory CD4+ T cells (Fig. **9F**). The results indicate the presence of different immune microenvironmental patterns, as well as of different cell-cell interaction networks between AS and CKD. We additionally carried out a post hoc exploratory analysis of the association of TAGLN with immune cell composition. TAGLN exhibited disease-specific immune correlation patterns. In the AS data, it positively correlated with naive B cells and M1 macrophages and negatively with Tregs and M0 macrophages; notably, a consistent negative correlation with M0 macrophages was the only immune association shared between AS and CKD (Fig. **9G**, **H**). This overlapping co-occurrence may indicate that TAGLN is associated with the presence of M0 macrophages in both these diseases, meriting future study of their biological significance.

### 3.8 Identification of Potential Candidate Drugs

The DGIdb was interrogated to identify potential therapeutics targeting the diagnostic gene, using default parameters. This analysis identified two candidate drugs: recombinant transforming growth factor-β1 (Combined Score = 2.48) and azacitidine (Combined Score = 1.3).

## 4 DISCUSSION

AS commonly coexists in patients with CKD. AS, particularly manifesting as CAD, is an important intermediary for the association of CKD to increased CV morbidity and mortality. Therefore, identifying CKD-AS coexpressed genes, which are crucial for understanding common pathogenesis between CKD and AS, has great scientific significance as CKD may accelerate the progression of AS and increase the incidence rate of cardia event [48]. Several mechanisms have been identified as responsible for this interaction, such as the build-up of uremic toxins, persistent inflammation, dysregulated lipid and electrolyte metabolism, NO deficiency, increased ROS production, and DNA and mitochondrial injury [49]. By applying bioinformatics methods in this study, we further clarify those complicated relationships, elucidate the pathogenesis behind the comorbidity between CKD and AS, and provide clues to the development of new therapeutics.

Integrative bioinformatics analyses identified 119 overlapping genes between AS and CKD through differential expression profiling and WGCNA. Functional enrichment analysis revealed convergent pathogenic mechanisms, including cytoskeletal dynamics in muscle cells, vascular smooth muscle contraction, and dysregulation of the cGMP-PKG and NF-κB signaling pathways. Vascular diseases caused by atherosclerosis, of which the key element is the alteration in VSMC phenotype, are one of the largest burdens to world health. Interestingly, mitochondria-cytoskeleton interactions are spatiotemporally regulating VSMC plasticity in cardiovascular disease [50]. Hypertension, a pivotal risk factor for CKD and heart failure, is underpinned by elevated vascular resistance due to arterial remodeling and hypercontractility, mediated through interconnected systems such as the renin-angiotensin-aldosterone axis, sympathetic hyperactivity, immune dysregulation, and oxidative stress [51]. Preclinical studies demonstrated that KUP-1 attenuates chronic renal failure (CRF)-associated vascular calcification and renal dysfunction *via* NO/cGMP/PKG pathway activation, concurrently reducing calcified nodule formation, calcium deposition, and alkaline phosphatase (ALP) activity in VSMCs [52]. Concomitantly, PNP functionalized scaffolds with polylactide-co-glycolide/rapamycin enhanced the adhesion of ECs and inhibited that of VSMCs *in vitro*, *in vivo*, these nano- coatings accelerated the re- endothelialisation, and had immunomodulatory effects *via* cGMP-PKG/NF-κB signalling, modulating both the biosynthetic cofactors as well as the immune response [53]. Taken together, these observations describe a multifactorial pathological axis connecting AS to CKD by genetic, molecular, and imunologîical crosstalk. Based on PPI network analysis *via* the CytoHubba of Cytoscape software, we found that there are 6 hub genes, and then selected LASSO regression, RF, and SVM-RFE—revealed together one of the most important diagnostic biomarkers for the co- occurrence of AS and CKD: TAGLN.ROC curve analysis confirmed its strong diagnostic performance. Transgelin (TAGLN, also known as SM22α), an actin-binding protein highly enriched in smooth muscle cells (SMCs), is a hallmark marker of SMC differentiation and the contractile phenotype. There are emerging data showing that this SMC phenotypic plasticity needed in vascular growth and healing can also be involved in atherogenesis and plaque instability [54]. Besides their structural functions, TAGLN contributes to angiogenesis *via* an elongation-dependent expression in endothelial cells [55]. CKD has been shown through transcriptome-wide studies of differential gene expression that is linked by a network of proteins between SOST and WNT5A to TAGLN *via* the transcription factor RUNX2 [56]. Proteomic profiling of human renal tubules and 5/6 nephrectomy rat models identified TAGLN as a novel biomarker related to renal fibrosis in CKD, mechanistically associated with the mitigation of mitochondrial dysfunction and oxidative stress [57]. Intriguingly, however, in the model of puromycin-induced nephropathy, dynamic cytoskeletal reorganization of podocytes has been described, including time-upregulated TAGLN in nine remodeled structure genes [58]. In this work, we present TAGLN as a two-sided diagnosis biomarker of CKD-AS comorbidity, linking cardiovascular and kidney disease through modulation of the actin cytoskeleton and cell response to oxidative stress.

AS and CKD have many common pathological characteristics, such as the accumulation of extracellular matrix (ECM), inflammation activation, phenotypic cell transition, and fibrosis [59, 60], which suggests that there are shared underlying pathological processes. With regard to the biological functions identified through our bioinformatic analyses and their clear overrepresentation in terms of actin filament organization-related processes, we postulate the following common hypothesis: aberrant regulation of cytoskeletal dynamics is an important, shared pathogenetic mechanism that links vascular remodeling in AS to renal fibrosis in CKD. In physiologic conditions, vascular smooth muscle cells (VSMCs): mainly contractile phenotype. Nevertheless, following prolonged stimulation by pathological signals, the VSMC changes its phenotype toward a more synthetic one with decreased expression levels for contractile markers and increased expression levels for synthetic markers like OPN or MMP2, which leads to enhanced cell migration and proliferation at the expense of contractility [61, 62]. While a controlled synthetic phenotype may contribute to vascular repair, its dysregulation promotes the development of AS [63]. Growing evidence indicates that cytoskeletal proteins are involved in regulating cell death processes, including apoptosis and pyroptosis, which are closely associated with the pathogenesis of kidney disease [64]. Hence, targeting the main players in the regulation of the actin cytoskeleton turnover could be an attractive option to treat kidney disease. In particular, Transgelin(TAGLN)—as an important regulator of the structure and dynamic behavior of actin, it is highly expressed in smooth muscle and contractile cells; therefore, TAGLN may be one of the most important targets involved in the comorbidity between AS and CKD.

Abnormal immune cell activation might contribute to the immune dysregulation of CKD or AS. In our comparative immune infiltration, we found some similarities between these two diseases, including an increase in the number of M0 macrophages and a decrease in the number of resting memory CD4+ T cells. The polarisation of M1 macrophages in the kidney may promote inflammatory injury by causing a cytokine storm. In previous work, it has been demonstrated that the inhibition of the STING pathway (C-176) could reduce LPS-mediated AKI through suppression of macrophage migration and downregulation of M1 polarization [65]. Likewise, atherogenesis seems dependent on macrophage foam cell formation and inflammatory polarization with IFN-γ/LPS-activated M1 macrophages playing the major role in early plaque progression [66, 67]. The pathophysiology of vascular-renal crosstalk is also contributed to by T-cell-mediated immunity. The reduced number of resting memory CD4+ cells in the advanced atherosclerotic plaque is also reflected in our CKD-AS setting [68], and animal models also support that T cells play a central role in atherogenic immune response [69, 70]; however, it is still to be clarified how resting memory CD4+ T cells contribute to the CKD-AS comorbid condition mechanistically. The correlation results showed an inverse relationship between M0 macrophages invasion and TAGLN expression, which suggested that TAGLN might be involved in CKD-AS development by regulating immunity, and positions it as a candidate drug target to block the common pathway of diseases.

DGIdb was used to perform drug-gene interactions, which yielded two potential candidates for treating TAGLN, including recombinant transforming growth factor-β1 (TGF-β1) and azacitidine. Among them, TGF-β1 can regulate the expression of multiple genes, including smooth muscle cell- specific genes and extracellular matrix protein genes, which play an important role not only during vasculogenesis but also in the pathogenesis of vascular disease [71]. Conventionally, TGF-β1 is considered a potent pro-fibrotic factor leading to excessive accumulation of extracellular matrix [72]. Several prior reports showed that TGF-β mRNA and protein were expressed at low levels in normal arteries; their expression increased in vascular cells and infiltrating leukocytes after injury or during atherogenesis [73-75]. However, this work suggests that TGF-β1 could be an agent of anti-AS, anti-CKD effect through the regulation of TAGLN. Though it seems contradictory to traditional belief, existing evidence suggests that loss of TGF-β signaling exacerbates atherosclerosis [76-78], and increased TGF-β expression in lesioned arteries is regarded as a protective mechanism [79]. Nevertheless, TGF-β1 is still an important inducer for kidney injury and initiates several pathological processes in renal cells, especially mesangial cells [80]. In the course of CKD, TGF-β1 mediates inflammatory and fibrotic signal transduction through the transcription factor Smad3 [81]. Therefore, this specific prediction might not directly suggest TGF-β1 as a therapeutic agent, but rather reinforces the central role of the TGF-β/TAGLN axis in the shared pathophysiology of AS and CKD. Intervention targeting components upstream or downstream of this pathway might represent a more feasible strategy. In contrast, the identification of azacitidine presents a more direct and novel therapeutic hypothesis. The DNA methyltransferase inhibitor azacitidine is an effective drug used in “demethylating therapy” [82]. As an epigenetic agent, it has become one of the promising strategies in treating abnormal angiogenesis [83]. Moreover, growing evidence suggests that epigenetic regulation is involved in the occurrence and development of renal fibrosis, and DNA demethylation may be an effective therapeutic approach to treat renal fibrosis [84]. These results open up new avenues in tackling the challenging comorbidity of AS and CKD. However, there are still some limitations in this work. Firstly, we used CIBERSORT to perform the immune infiltration analysis, a computational deconvolution technique that relies upon the accuracy of its reference signatures and linear assumptions. Applied to bulk transcriptomic data obtained in heterogeneous and inflamed tissue, these conditions are not necessarily met and thus could impact the accuracy in estimating proportions per cell type. Secondly, there is an unbalanced ratio between cases and controls in the CKD data set (GSE66494) (53 *vs.* 8), but we have not performed any re-sampling technique like SMOTE. We addressed it by applying the multi-method feature selection procedure (Lasso, RF, and SVM-RFE) for increasing its robustness, as well as validating our final gene signature on independent balanced cohorts. Despite that, the imbalanced nature of the training set is also an obstacle for the generality of the intermediary analysis steps. Most importantly, these results need to be validated experimentally by *in vivo*/*in vitro* functional experiments.

## 5 STUDY LIMITATIONS

First, this study is constrained by the absence of dedicated empirical experiments to validate the findings; hence, the reliability of relevant conclusions requires further experimental confirmation in future research. Second, the relatively limited sample scale may introduce potential sampling bias, which could partly restrict the representativeness and generalizability of the obtained results.

## CONCLUSION

In this study, we have identified TAGLN as a strong diagnostic biomarker for the comorbidity of AS and CKD by using the integrated bioinformatics analysis. Combined with multiple MLs, we showed that TAGLN has excellent diagnostic ability in 6 independent data sets, and furthermore, our results show, for the first time, that cytoskeleton deregulation could be a common pathway connecting vascular disease in AS and kidney fibrosis in CKD. The immune infiltration showed that the expression of TAGLN was negatively correlated with M0 macrophage, which suggested that TAGLN participated in the immunomodulation process. The drug prediction demonstrated that TGF- β1 and azacitidine might have clinical efficacy by regulating TAGLN. All these results elevated the status of TAGLN from a diagnostic indicator to a treatment target, providing new insights into the common pathogenesis and intervention of AS and CKD comorbidly.

## Figures and Tables

**Fig. (1):**
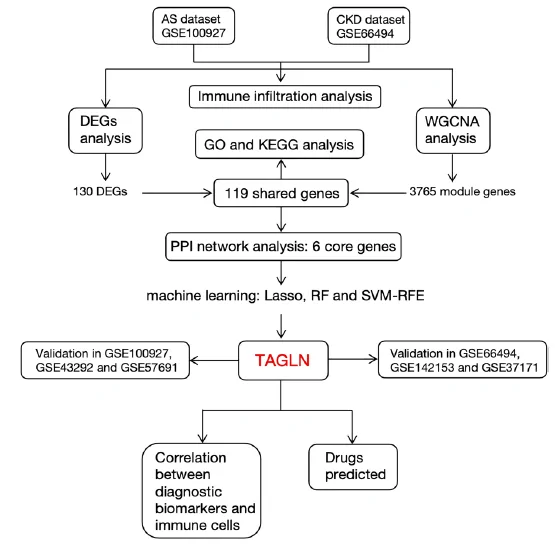
Detailed flowchart of research design.

**Fig. (2):**
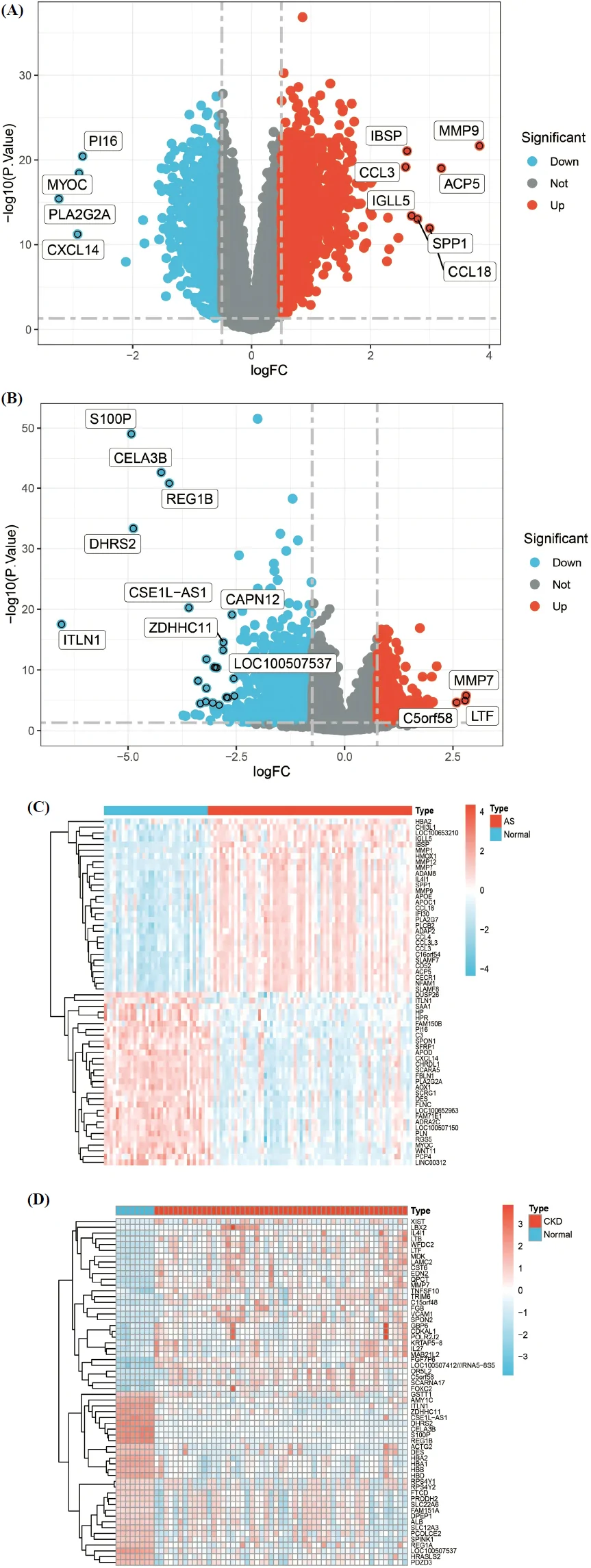
Visualization results of differential analyses of GSE100927(AS) and GSE66494(CKD). (**A**) The volcano plot of DEGs for AS. (**B)** The volcano plot of DEGs for CKD. In the volcano plots, up-regulated genes are shown in red, down-regulated genes are shown in blue, and genes with no significant difference are shown in grey. (**C**) The heatmap of the top 30 DEGs for AS. (**D**) The heatmap of the top 30 DEGs for CKD.

**Fig. (3):**
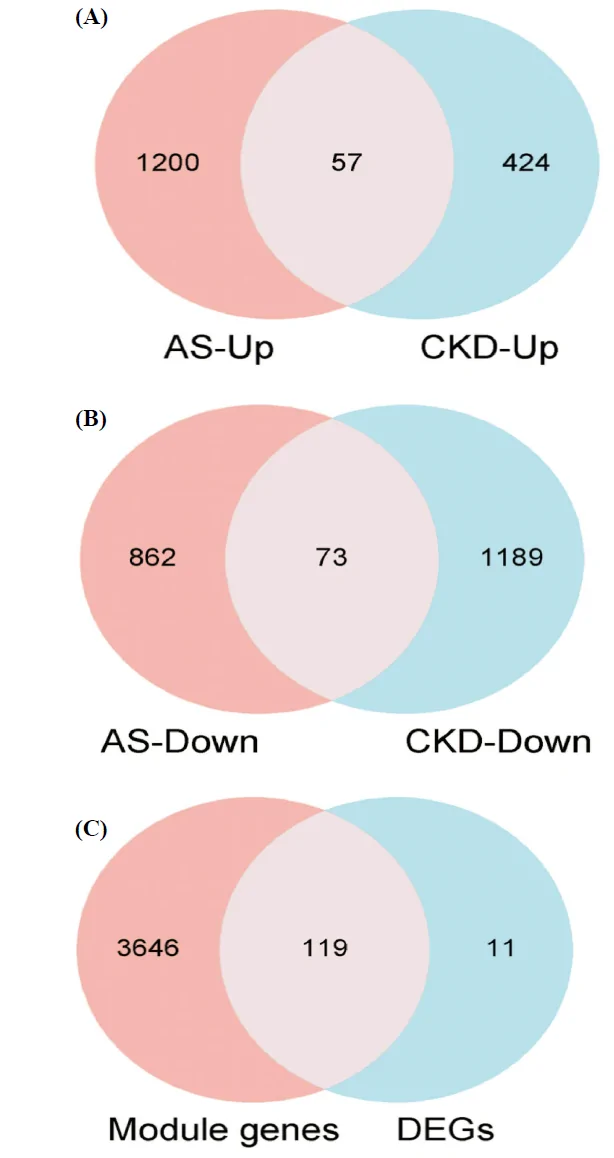
Venn diagrams. (**A)** total of 57 up-regulated genes between CKD and AS. (**B)** 73 down-regulated genes between CKD and AS. (**C**) 119 overlapped shared genes between the “Module genes” and “DEGs” datasets.

**Fig. (4):**
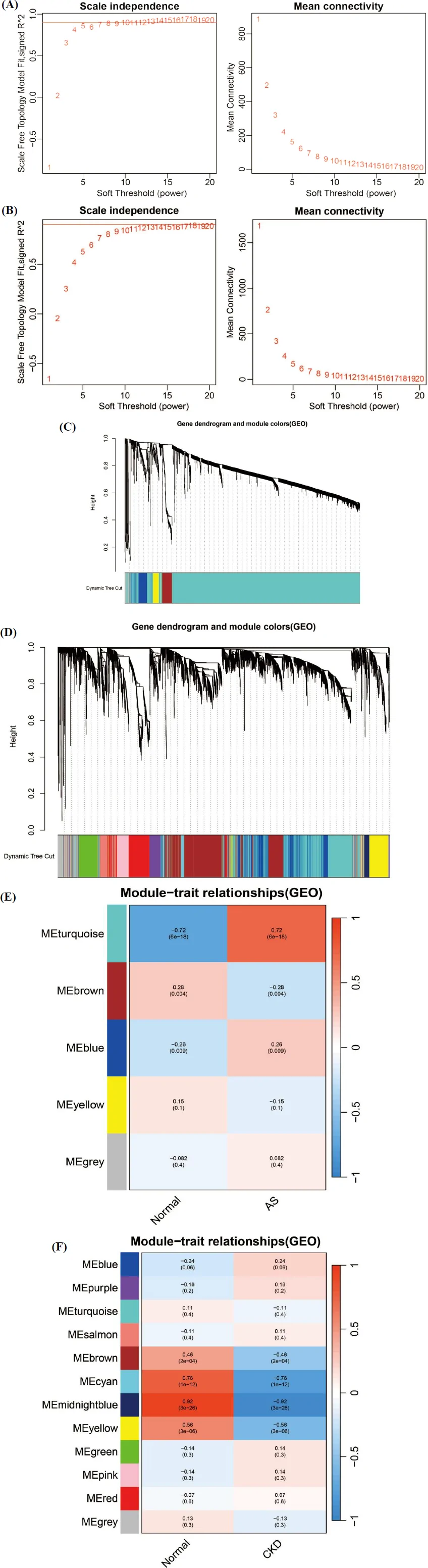
WGCNA. (**A**) The selection of soft threshold in AS (GSE100927). (**B**) The selection of soft threshold in CKD (GSE66494). (**C**) The cluster dendrogram of co-expression genes for AS (GSE100927). (**D**) The cluster dendrogram of co-expression genes for CKD (GSE66494). (**E**) Module-trait relationships for AS (GSE100927). (**F)** Module-trait relationships for CKD (GSE66494).

**Fig. (5):**
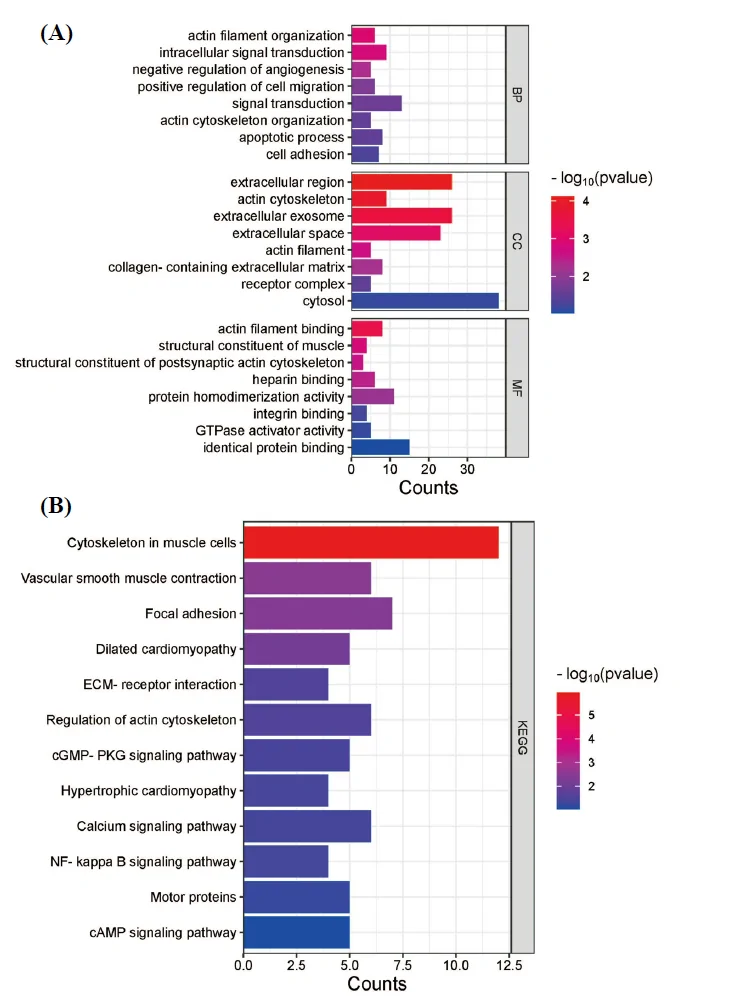
GO and KEGG enrichment analysis. (**A**) The top 8 GO functions. (**B**) The top 12 KEGG pathways.

**Fig. (6):**
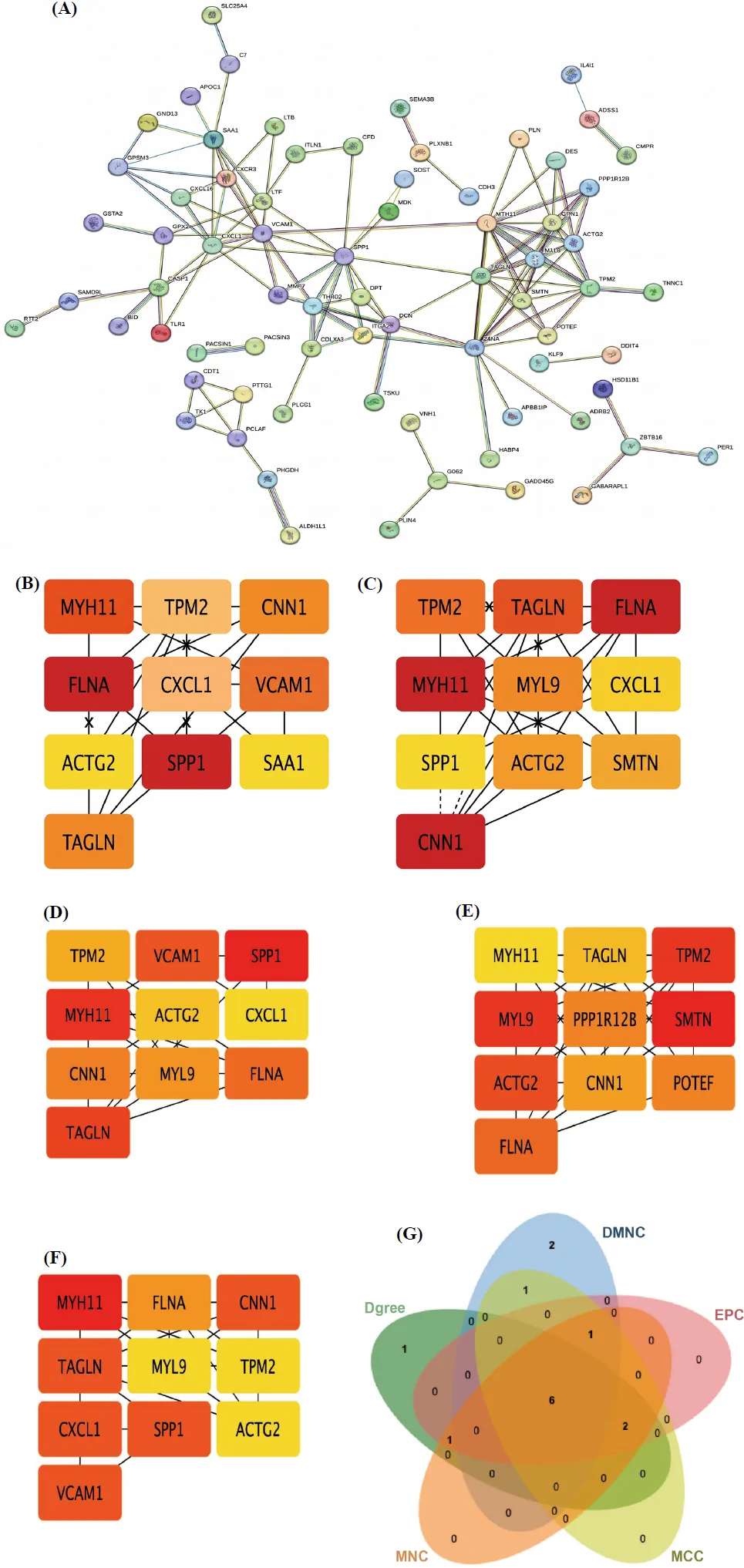
Core genes identification. (**A**) PPI network (confidence > 0.4). (**B**) The top 10 genes of the Degree algorithm. (**C**) The top 10 genes of the MCC algorithm. (**D**) The top 10 genes of the EPC algorithm. (**E**) The top 10 genes of the DMNC algorithm. (**F**) The top 10 genes of the MNC algorithm. (**G**) The overlaps of five algorithms.

**Fig. (7):**
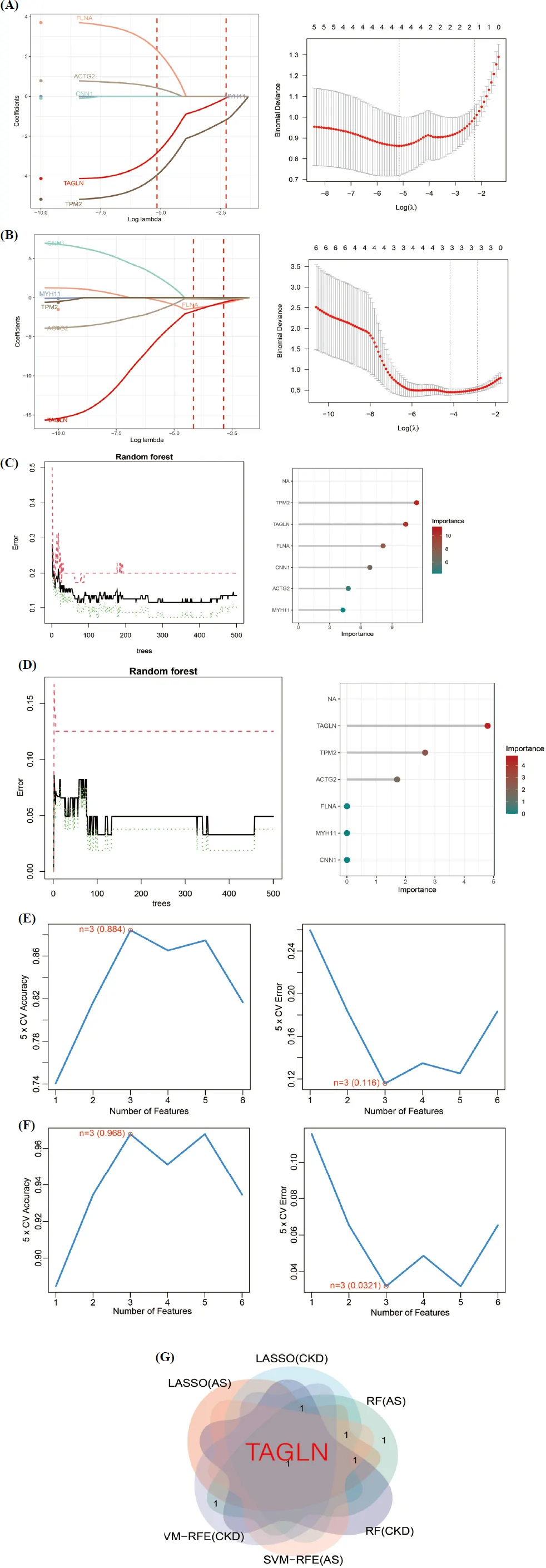
Machine learning-based selection of feature genes. (**A**, **B**) LASSO regression (10-fold cross-validation) selected 4 (AS) and 3 (CKD) genes. (**C**, **D**) RF identified 6 (AS) and 3 (CKD) key genes. (**E**, **F**) SVM-RFE determined the optimal 3- gene combination for AS (accuracy=0.884, RMSE=0.116) and CKD (accuracy=0.968, RMSE=0.032). (**G**) Intersection analysis identified TAGLN as the shared key gene.

**Fig. (8):**
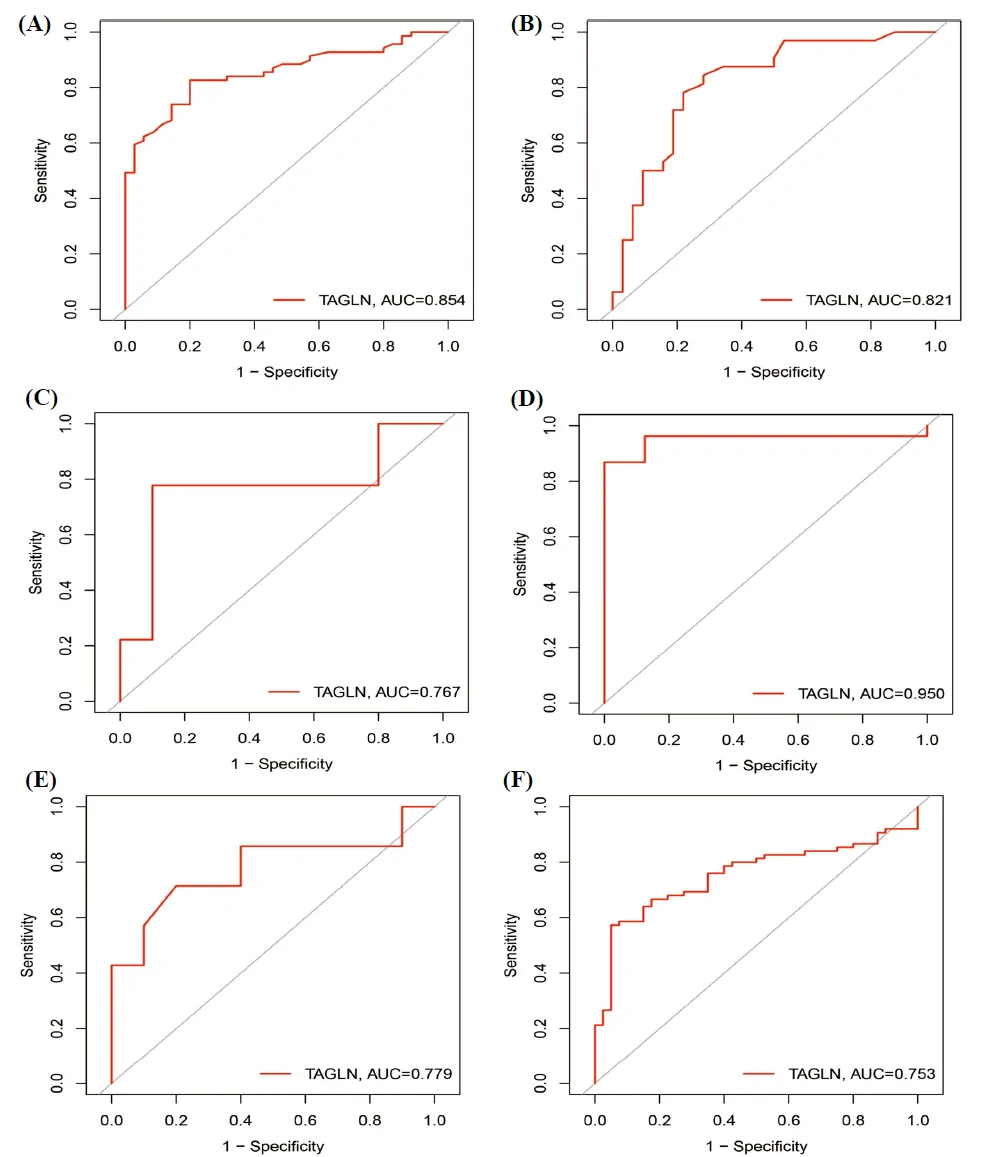
The diagnostic values of TAGLN in training and validation datasets. (**A-C**) ROC graphs of TAGLN in AS datasets (GSE100927, GSE43292, and GSE57691). (**D-F**) ROC graphs of TAGLN in CKD datasets (GSE66494, GSE142153, and GSE37171).

**Fig. (9):**
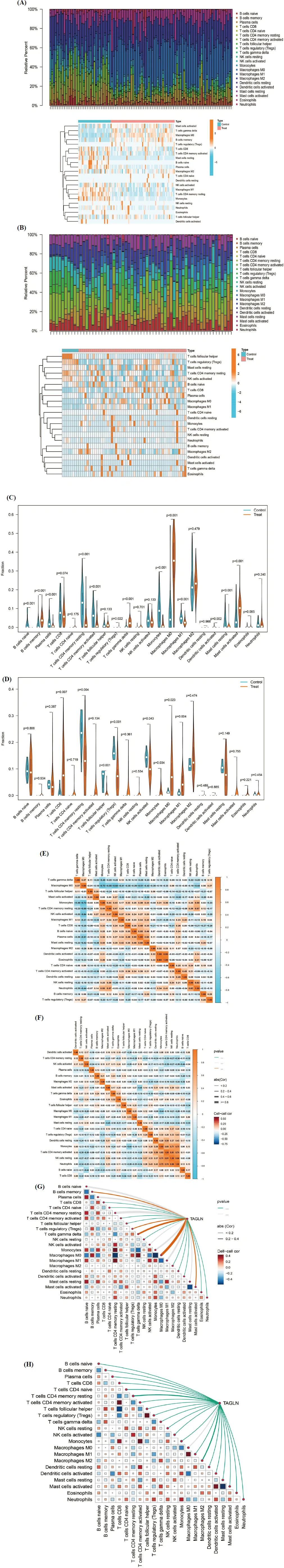
Assessment of immune cell infiltration. (**A**) The barplot and heatmap display the infiltration levels of immune cells in AS. (**B**) The barplot and heatmap display the infiltration levels of immune cells in CKD. (**C**, **D**) The violin plots comparing the proportion of immune cells between disease and control for AS and CKD. (**E**, **F**) The heatmaps depict the correlations among various immune cell types in AS and CKD. (**G**, **H**) Correlational analysis of immune cell types with TAGLN for AS and CKD.

## Data Availability

Datasets used in this study were downloaded from the Gene Expression Omnibus database under accession number GSE100927, GSE66494, GSE43292, GSE142153, GSE57691, and GSE37171 and are available at the following URLs: https://www.ncbi.nlm.nih.gov/geo/query/acc.cgi?acc=GSE100927, https://www.ncbi.nlm.nih.gov/geo/query/acc.cgi?acc=GSE66494, https://www.ncbi.nlm.nih.gov/geo/query/acc.cgi?acc=GSE43292, https://www.ncbi.nlm.nih.gov/geo/query/acc.cgi?acc=GSE142153, https://www.ncbi.nlm.nih.gov/geo/query/acc.cgi?acc=GSE57691, and https://www.ncbi.nlm.nih.gov/geo/query/acc.cgi?acc=GSE37171.

## LIST OF ABBREVIATIONS

AS: Atherosclerosis
CKD: Chronic Kidney Disease
CVDs: Cardiovascular Diseases
ESRD: End-Stage Renal Disease
GEO: Gene Expression Omnibus
DEGs: Differentially Expressed Genes
WGCNA: Weighted Gene Co-Expression Network Analysis
GO: Gene Ontology
KEGG: Kyoto Encyclopedia of Genes and Genomes
PPI: Protein–Protein Interaction
LASSO: Least Absolute Shrinkage and Selection Operator
RF: Random Forest
SVM-RFE: Support Vector Machine-Recursive Feature Elimination
ROC: Receiver Operating Characteristic
AUC: Area Under Curve
CIBERSORT: Cell Type Identification By Estimating Relative Subsets Of RNA Transcripts
DGIdb: Drug-Gene Interaction Database
TOM: Topological Overlap Matrix
MEs: Module Eigengenes
STRING: Search Tool for the Retrieval of Interacting Genes/Proteins
MCC: Maximal Clique Centrality
DMNC: Density of Maximum Neighborhood Component
EPC: Edge Percolated Component
MNC: Maximum Neighborhood Component
MDG: Mean Decrease of Gini index
TAGLN: Transgelin
VSMCs: Vascular Smooth Muscle Cells
cGMP-PKG: Cyclic Guanosine Monophosphate-Protein Kinase G
NF-κB: Nuclear Factor-kappa B
Treg: Regulatory T Cell
NK: Natural Killer
DCs: Dendritic Cells
TGF-β1: Transforming Growth Factor-β1
ECM: Extracellular Matrix
NO: Nitric Oxide
ROS: Reactive Oxygen Species
